# Korea Nurses’ Health Study and the health of reproductive-aged women: a cohort profile

**DOI:** 10.4178/epih.e2024048

**Published:** 2024-04-30

**Authors:** Chiyoung Cha, Heeja Jung, Sue Kim, Jung Eun Lee, Kwang-Pil Ko, Eunyoung Cho, Hyun-Young Park, Joong-Yeon Lim, Bo Mi Song, Sihan Song, Soojin Park, Aram Cho

**Affiliations:** 1 College of Nursing, Ewha Research Institute of Nursing Science, Graduate Program in System Health Science and Engineering, Ewha Womans University, Seoul, Korea; 2 College of Nursing, Konyang University, Daejeon, Korea; 3 College of Nursing, Yonsei University, Seoul, Korea; 4 Department of Food and Nutrition, College of Human Ecology, Research Institute of Human Ecology, Seoul National University, Seoul, Korea; 5 Clinical Preventive Medicine Center, Seoul National University Bundang Hospital, Seongnam, Korea; 6 Department of Dermatology, The Warren Alpert Medical School of Brown University, Providence, RI, USA; 7 Korea National Institute of Health, Cheongju, Korea; 8 Division of Population Health Research, Department of Precision Medicine, Korea National Institute of Health, Cheongju, Korea; 9 College of Nursing, Graduate Program in System Health Science and Engineering, Ewha Womans University, Seoul, Korea

**Keywords:** Cohort studies, Women, Nurses, Women’s health

## Abstract

The Korea Nurses’ Health Study (KNHS) is an ongoing, large-scale, prospective cohort study of women nurses, focusing on the effects of occupational, environmental, and lifestyle factors on the health of women. The first KNHS survey was performed in 2013-2014 (n=20,613). As of December 2023, 11 follow-up surveys have been conducted. Participants who were pregnant were asked to participate in the early pregnancy survey (n=2,179) and postpartum survey after giving birth (n=2,790). The main variables included socio-demographic, work-related, lifestyle, physical, mental, and women’s health factors. Blood, urine, and toenail samples were collected from a participant subgroup of the first survey (n=1,983). The subgroups of the second survey completed a food frequency questionnaire in 2019 (n=300) and 2021 (n=871). In 2020, a subgroup of the first survey answered a coronavirus disease 2019-related survey (n=975). To examine various health-related factors in young adults, new participants were added to the KNHS cohort in the 11th (n=1,000) and 12th (n=1,002) surveys. The KNHS cohort will help identify health and illness determinants in Korean women. Data can be accessed at https://coda.nih.go.kr/frt/index.do.

## INTRODUCTION

Although the life expectancy of women is gradually increasing [1], their health-related quality of life and global burden of diseases have not improved [2,3]. The increasing gap between life expectancy and a healthy life expectancy is a greater issue in women than in men [4,5]. This is especially true in Korea where chronic diseases increase the health burden of women. For example, the mortality rate for hypertension-related deaths is higher in women (3.5%) than in men (1.4%) [6]. Furthermore, the prevalence of chronic diseases, including osteoporosis, hypercholesterolemia, anemia, and allergic rhinitis, is higher in women than in men [6,7]. Moreover, the lack of disease predictors for women’s health leads to increased healthcare costs [8,9]. Further studies are needed to better understand the health determinants in women.

Accordingly, research projects based on communities of women have been conducted in Western countries to generate knowledge and evidence for medical treatments and health policies. The Women’s Health Initiative project in the United States, which investigated cardiovascular diseases, breast and colon cancers, and osteoporosis in menopausal women [10], changed the global hormone therapy standards for menopausal women [11]. The Australian Longitudinal Study on Women’s Health provides evidence for the development of women’s health, welfare, and healthcare service policies. It has been used to develop physical activity and sedentary behavior guidelines tailored to adults and elderly people, outlining novel considerations for physical activity. Furthermore, these guidelines served as the basis for the 2017 perinatal mental healthcare guidelines [12].

When studying women’s health, women nurses have been considered exceptional participants because they are able to provide accurate health and illness information [13]. One classic example is the Nurses’ Health Study (NHS), which was conducted in the United States in 1976 to survey the lifestyles, working environments, and health of women [14]. The early NHS finding of a 97% breast cancer prevalence was comparable to that of the National Cancer Institute’s Surveillance, Epidemiology, and End Results Program, demonstrating the generalizability of the study [15]. During the past 40 years, successive NHSs (NHS, NHSII, and NHS3) have provided significant information and evidence for women’s health [14,16,17].

This has been especially important in Korea because the fertility rate in Korea has dropped to 0.78 (births per woman) [18], the lowest among the Organization for Economic Cooperation and Development (OECD) countries [19]. In addition, Korea has the highest suicide rate of women among OECD countries [20]. Although several studies have generated new knowledge regarding women’s health [21-23], further longitudinal data are needed to study the long-term effects of social, cultural, demographic, and physiological differences on women’s health. In addition, most health cohort studies that include women in Korea have been focused on specific patient groups or limited to certain age ranges such as perimenopause [24]. Collecting longitudinal data on reproductive-aged women would yield valuable information on the health and illness determinants of Korean women; thus, the Korea Nurses’ Health Study (KNHS) was implemented [25].

Data from the KNHS’s baseline survey were opened to the public in September 2023, and successive follow-up survey data will also be opened to the public. Accordingly, a cohort profile paper will help scholars who plan to use these data for analysis.

### Purpose

The present study introduced the KNHS, a prospective cohort study that focuses on the health of reproductive-aged women. The specific aims of this study were to describe the baseline and follow-up surveys conducted over the past decade and to summarize the key findings.

## STUDY PARTICIPANTS

### Design and participants

**Figure 1.**
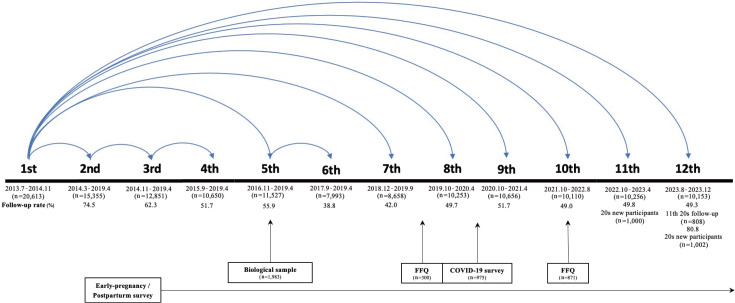
Design of the Korea Nurses’ Health Study. The blue line means eligible participants for each survey. FFQ, food frequency questionnaire; COVID-19, coronavirus disease 2019.

The KNHS was launched in 2013 and is currently in its 12th survey (Figure 1). The items and structure of this cohort study were based on the NHS3 in the United States. A total of 20,613 women registered nurses (reproductive ages 20 to 45 years) working at hospitals were recruited for the baseline survey using a simple random sampling strategy [25].

Except for the third, fourth, and sixth surveys, which followed up on participants from the previous surveys, all follow-up surveys followed up on participants in the first survey. In the second survey, the follow-up rate was 74.5%, which gradually decreased to 51.7% in the fourth survey. Until the fourth survey, the participants from the previous surveys waited for 6 months to participate in the follow-up surveys. However, because of the increasing attrition rate, participants from the baseline survey have been eligible, since the fifth survey in 2016, to participate in follow-up surveys without any restrictions on the resting period between surveys. Moreover, starting from the second survey onwards, only participants who had participated in the previous survey were eligible, while in the fifth follow-up survey, participants from the baseline survey were eligible. Consequently, the follow-up rate in the fifth survey increased slightly to 55.9%. However, the followup rate in the sixth survey markedly decreased to 38.8% because only participants in the previous survey were allowed to participate. Because baseline participants were enabled to join the seventh survey, the follow-up rate in the seventh survey reached 42.0%. Since the eighth survey, a follow-up rate of approximately 50% has been maintained (Figure 1). The follow-up rate for the 11th survey was 49.8%. We found that participants who were younger and had lower education levels were less likely to respond to follow-up surveys.

Data collection for the baseline survey was conducted between July 2013 and November 2014 (n= 20,613). In the first phase, subsequent surveys were conducted at 6-month to 8-month intervals. Beginning with the fifth survey, surveys were conducted every year. To increase the retention rate, data collection for the second to sixth surveys was kept open until April 2019. However, analysis of the participation patterns showed that a longer data collection period did not increase the retention rate of participants. Thus, beginning with the seventh survey, the data collection period for each survey lasted for several months and was discontinued before collecting data for the next survey (i.e., participants were asked to complete the survey within the appropriate period). After the 10th survey, the proportion of women in their 20s decreased to 2.6% as the participants aged. For the 11th and 12th surveys, 2,002 women nurses in their 20s were recruited to compare changes in young adults and examine various health-related factors. The 12th survey was completed in December 2023.

The KNHS generated valuable data through the early pregnancy and postpartum surveys. Since 2014, participants who were pregnant during each survey were contacted for the early pregnancy survey and again for the postpartum survey after giving birth. The early pregnancy survey was administered between gestational weeks 20 and 25. In addition, the participants were asked to complete the postpartum survey 6 weeks after their estimated date of delivery. These surveys began in July 2014. As of July 2023, the early pregnancy and postpartum surveys included 2,179 and 2,790 participants, respectively.

Additional surveys of subgroups were conducted 4 times. In 2016, biological data, including blood, urine, and toenail samples, were provided by a subgroup of the first survey (n= 1,983). The subgroups of the second survey completed a food frequency questionnaire in 2019 (n= 300) and 2021 (n= 871). In 2020, a subgroup of the first survey answered a coronavirus disease 2019 (COVID-19)-related survey (n= 975) (Figure 1).

Since the beginning, the KNHS surveys have been conducted online with participants receiving text messages with links for each survey.

### Ethics statement

All surveys were reviewed and approved by institutional review boards (IRBs). Surveys 1 through 4 were approved by the IRB of the Korea Centers for Disease Control and Prevention (#2013-03CON-03-P), the funding agency. Surveys 5 through 11 were approved by the IRB at the principal investigator’s institution (12th survey IRB No. ewha-202208-0016-01). Before completing the surveys, participants were provided with an online information sheet. Participation in the online survey was considered voluntary, and participants were informed of their right to withdraw from the study at any time without any disadvantage.

## MEASUREMENTS

Key variables from the surveys are shown in Table 1. The baseline KNHS survey items were organized in accordance with the NHS3 survey items, following a memorandum of agreement between the NHS3 and KNHS researchers. The KNHS research team reviewed and consulted experts on the survey items before conducting data collection for each survey. As of the ninth survey, the variables have been based on the current questionnaire. Lifestyle items, including drinking, smoking, medication usage, and health screening, were assessed every other year to minimize the burden on participants. Previously developed scales were used to measure mental health variables. The Subjective Happiness Scale [26] and Life Orientation Test [27] were used to measure subjective happiness and optimism, respectively. Meanwhile, the Pittsburgh Sleep Quality Index [28] or the Jenkins Sleep Questionnaire [29] were used to measure sleep quality. The Perceived Stress Scale [30], Chalder Fatigue Scale [31], and Patient Health Questionnaire [32] were used to measure perceived stress, fatigue, and depressive symptoms, respectively. The State-Trait Anxiety Inventory [33] or the Generalized Anxiety Disorder 7-item scale [34] was used to measure anxiety. Physical and psychological health, work environment, and lifestyle factors (e.g., eating habits, physical activity, drinking, smoking, and intake of supplements and medications) were included as survey items for early pregnancy. Questions regarding pregnancy, physical and psychological health, and lifestyle after giving birth were included as survey items for postpartum women (Table 2).

## KEY FINDINGS

### Demographic characteristics

The demographic characteristics of participants in surveys 1 (baseline) to 11 are shown in Table 3. In the baseline survey, most participants were in their 20s (58.5%), and the mean age was 29.39± 5.92 years. In the 11th survey, most participants were in their 30s (62.4%), and the mean age was 38.32± 5.98 years. In the baseline survey, 65.7% of participants were single, whereas most participants (72.6%) were married in the 11th survey. The percentage of participants with a bachelor’s degree increased from 45.2% to 62.7% and those with master’s degrees or PhDs increased from 7.4% to 20.9% in the baseline and 11th surveys, respectively. A high proportion of graduate school students could be attributed to advanced practice nursing programs, which are master’s degree programs [35]. The mean body mass index was 20.94 kg/m² and 22.62 kg/m² in the baseline and 11th surveys, respectively. Approximately 81.0% of participants in the 11th survey were working, with 83.1% of those working in healthcare. Comparing the baseline and 11th surveys, approximately 83.0% and 71.0% of participants drank alcohol during the past year, and 1.0% and 0.6% smoked, respectively.

### Diagnosed diseases

The prevalence of the most frequently diagnosed diseases and symptoms is shown in Table 4. Disease diagnosis was not surveyed in the second and sixth surveys. In the baseline survey, the most frequently diagnosed disease was allergic rhinitis (19.5%), followed by gastritis/gastric ulcer/duodenal ulcer (19.1%), gastroesophageal reflux disease (15.0%), and eczema/atopic dermatitis (11.6%). Although the pattern of frequently diagnosed diseases was maintained in the progression of surveys, their prevalence increased.

The prevalence of thyroid cancer was 3 times higher than cervical cancer, which was the second most prevalent. This pattern continued until the 11th survey when breast cancer overtook cervical cancer as the second most prevalent.

### Demographic characteristics from the early pregnancy and postpartum surveys

Demographic characteristics from the early pregnancy and postpartum surveys are presented in Table 5. The mean age of participants in the early pregnancy and postpartum surveys was 32.07± 3.68 years and 32.64± 3.82 years, respectively. Only 0.1% of participants who were pregnant smoked, whereas 0.4% of postpartum participants smoked. In terms of supplement intake, pregnant participants took folic acid most frequently (86.5%), followed by iron (40.5%), fish oil (13.4%), beta-carotene (4.8%), and flaxseed or flaxseed oil (3.2%). Four out of 5 pregnant participants (80.8%) were working. Among them, 47.7% were working in shifts, with 41.4% working the night shift. The mean sleep time for pregnant and postpartum participants was 7.28 hours and 6.36 hours per day, respectively. Approximately 57.2% delivered their baby vaginally and 41.1% via cesarean section. During pregnancy, the mean postnatal depression score was 10.30± 5.67, with a score ≥ 10 indicating clinical depression symptoms [36]. The mean postpartum depression score was 9.29± 6.43, indicating borderline levels of depressed mood.

## STRENGTHS AND WEAKNESSES

A unique feature of the KNHS is that it was designed to investigate health and illness determinants among healthy reproductive-aged women nurses over time using online surveys. We would like to discuss the strengths and limitations of the KNHS.

First, the most unique feature of the KNHS is that participation was limited to registered nurses. By using health professionals as participants in a health cohort study, accurate health and illness information based on professional knowledge could be collected. Moreover, because the work environment of nurses varies across a wide spectrum (e.g., rotating vs. fixed hours, labor-intensive units vs. administration), the role of various healthcare work environments could be explored [37-40]. Nevertheless, using health professionals as participants could present healthier behaviors. Thus, the results should be applied with caution when making inferences to the general population. For example, when compared to national data on Korean women, the participants in the 11th survey had higher education levels (bachelor’s degree or higher: 40.6 vs. 83.6%), were more likely to be working (67.9 vs. 81.0%), and reported a lower obesity rate (26.9 vs. 20.6%) [6,41]. Global studies on nurses have also demonstrated that nurses experience elevated job intensity and stress levels that influence their mental and physical health [42,43]. Although the prevalence of common diseases and health issues was comparable to that found in the general population of Korean women [6], these factors should be considered in the interpretation.

Another unique feature of the KNHS is that it was the only cohort study of healthy reproductive-aged women in Korea. Most health cohorts target specific populations with certain diagnoses or health issues (e.g., cancer or acquired immune deficiency syndrome) or specific age groups (e.g., elderly or menopausal women). The KNHS data identifies valuable information on young women’s health, which has been understudied. For example, in a previous study comparing the rate of postpartum depression in nurses, Korean nurses reported a rate more than 10 times higher than their United States counterparts [44]. Follow-up surveys provide comprehensive data over time on women’s reproductive health, lifestyle, medication, diagnosis, and menopause. Thus, the KNHS data can be used to identify the long-term modifiable determinants of health throughout womens’ lifespans.

Third, the online survey design of the KNHS is usually considered a weakness in terms of attrition in cohort studies [45]. This might be partially true in cohort studies conducted over a short term. For example, from the baseline survey to the seventh survey, the response rate of the KNHS decreased by 42%. Thus, the KNHS research team used various tailored strategies, such as obtaining second contact information, having mobile messengers respond to participants’ queries 24/7, providing age-appropriate gifts for compensation after the survey (e.g., cosmetic mobile coupons for participants in their 20s and coffee mobile coupons for those in their 30s to 40s), and mass-produced advertising (e.g., promoting the KNHS through online nursing communities, Instagram, and Facebook; sending out posters; and presenting briefing sessions to hospitals throughout Korea). However, conducting follow-up surveys online did not appear to be a drawback in the long-term. The response rate of the KNHS did not decreased over time, ranging between 49.0% (11th survey) and 51.7% (12th survey). Moreover, quarantine or social distancing policies did not influence follow-up surveys during the COVID-19 pandemic, nor did our response rate drop during this period.

Another unique feature of the KNHS is that it can proactively respond to social issues. Survey items were reviewed and modified or added before conducting each survey. For example, elevated levels of toxic chemicals were reported in menstrual products in Korea in 2017, causing fear among menstruating women [46]. Survey items related to the use of menstrual products as well as psychosocial variables were added to the survey. The results showed that the rate of anxiety was higher than the perception of safety for all menstrual hygiene products. In addition, there was a low awareness of toxic shock syndrome [47]. Another example is COVID-19. The KNHS research team quickly added items related to COVID-19 to the survey, such as changes in work, wearing personal protective equipment, and psychological reactions toward the pandemic. Fear, anxiety, and depression increased among nurses who cared for COVID-19-positive patients, and the safety culture in hospitals was found to impact their mental health [48].

As the cohort aged, additional women in their 20s were included in the 11th and 12th surveys. After 10 years of follow-up, most participants in the KNHS were over 30 years old. Women in their 20s were added because different generations of women might have different lifestyles and varying levels of harmful or beneficial exposures that impact their health. For example, a study of 3 generations of nurses reported significant differences in characteristics, work-related experiences, and attitudes by generation [49]. Moreover, the use of a cohort study design not only allows the identification of long-term health determinants but also allows cross-sectional analyses. The health and well-being of young women are particularly important in Korea because the birth rate continues to decline to ultralow levels. Thus, accumulating data on young Korean women is more important than ever.

Future use of KNHS data could contribute to the development of health policies. Studies of nurses’ health in other countries have played a significant role in public health. For example, the NHS in the United States influences various aspects of public health by providing health prevention recommendations and dietary and physical activity guidelines to organizations (e.g., the American Cancer Society, American Heart Association, and World Health Organization) as well as providing guidance for cancer survivors [14]. The Danish NHS, which was initiated to investigate the impact of hormone therapy on women, has provided data on the risks of hormone therapy and cancer incidence in Danish women consistent with the Women’s Health Initiative in the United States [50]. KNHS data on lifestyle, physical activity, diet, health screening, and disease diagnosis among Korean women have been collected for over a decade. This data could be utilized to develop national policies that promote public health. For example, by tracking the trend of health screening rates, targeted promotional strategies could be used to increase the screening rate. Data on the changes in physical activity levels and diet during the pandemic and post-pandemic period could be used to develop healthy lifestyle guidelines for the next unexpected pandemic. The KNHS data can be used to delineate health determinants among Korean women across different age groups. Armed with this evidence, tailored intervention strategies could be devised for each age group, potentially yielding advancements in public health.

## Figures and Tables

**Table 1.** Summary of variables measured in the Korea Nurses’ Health Study (2013-2023)

Variables	1st	2nd	3rd	4th	5th	6th	7th	8th	9th	10th	11th	12th
Socio-demographics	o	o	o	o	o	o	o	o	o	o	o	o
Work-related characteristics												
Employment status	-	-	-	-	-	o	o	o	o	o	o	o
Occupational exposures	o	-	-	-	o	-	-	-	o	-	o	-
Work violence	-	-	-	-	-	o	-	-	-	-	-	-
Work-life balance	-	-	o	-	-	o	-	-	-	-	-	-
Lifestyle characteristics												
Drinking and smoking	o	-	-	-	o	-	-	o	o	-	o	-
Diet	-	o	-	-	-	-	-	o^1^	-	o^1^	-	-
Coffee consumption	-	-	-	-	o	-	-	-	o	-	-	-
Physical activity	-	-	o	-	-	-	-	o	o	o	o	-
Physical health												
Medical history	o	-	o	o	o	-	o	o	o	o	o	o
Family history	o	-	-	-	o	-	-	-	o	-	o	-
Health screening	-	-	o	-	-	-	-	o	o	-	o	-
Medication usage	o	-	-	-	-	o	-	o	o	-	o	-
Mental health												
Subjective health	o	-	-	-	o	-	-	o	o	o	o	o
Subjective happiness	-	-	-	-	-	o	-	-	-	o	-	-
Optimism	-	-	-	-	-	o	-	-	-	-	-	-
Quality of life	-	-	-	-	o	-	-	o	o	-	o	o
Sleep quality	o	-	-	-	o	o	o	o	o	o	o	o
Perceived stress	o	-	-	-	o	-	o	-	o	o	o	o
Fatigue	o	-	-	-	o	-	-	o	o	o	o	o
Depressive symptoms	o	-	-	-	o	o	o	o	o	o	o	o
Anxiety	o	-	-	-	o	o	o	-	o	-	o	o
Women’s health characteristics												
Menstruation	-	-	o	-	-	-	o	-	o	-	o	-
Usage of menstrual products	-	-	-	-	-	-	o	-	o	-	o	-
Lifetime pregnancy	o	-	-	-	-	-	o	o	o	-	o	-
Current pregnancy	o	o	o	o	o	o	o	o	o	o	o	o
Additional questions	-	-	-	o^2,3^	-	o^4^	-	-	o^5^	o^5^	-	-

1 Additional survey.

2 Weight management.

3 Health related lifestyle during teenage years.

4 Pets.

5 Coronavirus disease 2019 items.

**Table 2.** Variables measured in the early-pregnancy and postpartum surveys of the Korea Nurses’ Health Study

Variables	Early-pregnancy	Postpartum
Socio-demographic	o	o
Work-related characteristics		
Employment status	o	-
Occupational exposures	o	-
Turnover intention	-	o
Lifestyle		
Drinking and smoking	o	o
Diet	o	o
Coffee consumption	-	o
Physical activity	o	o
Physical health		
Medication usage	o	o
Morning sickness	o	
Mental health		
Perceived stress	o	-
Depressive symptoms	o	o
Subjective health	o	o
Fatigue	o	o
Anxiety	o	-
Sleep quality	o	o
Planned pregnancy	o	-
Vitamin or supplement intake	o	-
Disease during pregnancy	-	o
About baby	-	o
Pregnancy outcome	-	o
Current feeding	-	o

**Table 3.** Characteristics of participants in the Korea Nurses’ Health Study across 11 surveys (2013-2023)

	1st (n=20,613)	2nd (n=15,355)	3rd (n=12,851)	4th (n=10,650)	5th (n=11,527)	6th (n=7,993)	7th (n=8,658)	8th (n=10,253)	9th (n=10,656)	10th (n=10,110)	11th (n=10,256)^1^
Age (yr)	29.39±5.92	30.58±6.01	31.59±6.02	32.20±5.85	32.71±5.98	33.84±5.98	34.60±5.86	35.23±5.90	36.48±6.01	37.40±5.99	38.32±5.98
20-29	12,055 (58.5)	7,948 (51.8)	5,842 (45.5)	4,288 (40.3)	4,330 (37.6)	2,331 (29.2)	1,931 (22.3)	1,821 (17.8)	949 (8.9)	266 (2.6)	43 (0.4)
30-39	6,842 (33.2)	5,768 (37.6)	5,361 (41.7)	4,903 (46.0)	5,441 (47.2)	4,168 (52.1)	4,971 (57.4)	6,064 (59.1)	6,655 (62.5)	6,611 (65.4)	6,397 (62.4)
40-49	1,716 (8.3)	1,638 (10.7)	1,648 (12.8)	1,457 (13.7)	1,749 (15.2)	1,477 (18.5)	1,679 (19.4)	2,186 (21.3)	2,696 (25.3)	2,725 (27.0)	3,166 (30.9)
≥50	0 (0.0)	1 (0.0)	0 (0.0)	2 (0.0)	7 (0.1)	17 (0.2)	77 (0.9)	182 (1.8)	356 (3.3)	508 (5.0)	650 (6.3)
Marital status											
Never married	13,548 (65.7)	-	-	-	5,288 (45.9)	3,236 (40.5)	3,039 (35.1)	3,312 (32.3)	3,089 (29.0)	2,674 (26.4)	2,376 (23.2)
Married	6,965 (33.8)	-	-	-	6,127 (53.2)	4,635 (58.0)	5,472 (63.2)	6,748 (65.8)	7,353 (69.0)	7,197 (71.2)	7,442 (72.6)
Divorced/separated/widowed	98 (0.5)	-	-	-	109 (0.9)	121 (1.5)	147 (1.7)	193 (1.9)	214 (2.0)	239 (2.4)	438 (4.3)
Missing	2 (0.0)	-	-	-	3 (0.0)	1 (0.0)	0 (0.0)	0 (0.0)	0 (0.0)	0 (0.0)	0 (0.0)
Education (yr college)											
3	9,771 (47.4)	-	-	-	3,104 (26.9)	1,737 (21.7)	1,696 (19.6)	1,991 (19.4)	1,945 (18.3)	1,715 (17.0)	1,684 (16.4)
4	9,316 (45.2)	-	-	-	6,882 (59.7)	5,000 (62.6)	5,568 (64.3)	6,481 (63.2)	6,726 (63.1)	6,417 (63.5)	6,426 (62.7)
Master’s or doctoral degree	1,525 (7.4)	-	-	-	1,538 (13.4)	1,254 (15.7)	1,394 (16.1)	1,781 (17.4)	1,985 (18.7)	1,978 (19.6)	2,146 (20.9)
Missing	1 (0.0)	-	-	-	3 (0.0)	2 (0.0)	0 (0.0)	0 (0.0)	0 (0.0)	0 (0.0)	0 (0.0)
Body mass index (kg/m^2^)	20.94±2.72	-	-	-	21.82±3.15	22.01±3.02	22.21±3.26	22.27±3.28	22.51±3.36	22.50±3.35	22.62±3.45
Underweight (<18.5)	3,155 (15.3)	-	-	-	1,119 (9.7)	748 (9.4)	683 (7.9)	757 (7.4)	718 (6.7)	654 (6.5)	627 (6.1)
Normal (18.5-22.9)	13,490 (65.4)	-	-	-	7,089 (61.5)	4,745 (59.4)	5,062 (58.5)	6,005 (58.6)	5,978 (56.1)	5,740 (56.8)	5,685 (55.4)
Overweight (23.0-24.9)	2,167 (10.5)	-	-	-	1,641 (14.2)	1,232 (15.4)	1,413 (16.3)	1,727 (16.8)	1,860 (17.5)	1,740 (17.2)	1,792 (17.5)
Obese (≥25.0)	1,708 (8.3)	-	-	-	1,676 (14.5)	1,250 (15.6)	1,493 (17.2)	1,764 (17.2)	2,100 (19.7)	1,976 (19.5)	2,152 (21.0)
Missing	93 (0.5)	-	-	-	2 (0.0)	18 (0.2)	7 (0.1)	0 (0.0)	0 (0.0)	0 (0.0)	0 (0.0)
Work status											
Yes	20,613 (100)	-	-	-	-	7,260 (90.8)	7,667 (88.6)	9,010 (87.9)	8,885 (83.4)	8,328 (82.4)	8,309 (81.0)
No	-	-	-	-	-	731 (9.1)	991 (11.4)	1,242 (12.1)	1,771 (16.6)	1,782 (17.6)	1,947 (19.0)
Missing	-	-	-	-	-	2 (0.0)	0 (0.0)	1 (0.0)	0 (0.0)	0 (0.0)	0 (0.0)
Working in a health-care facility^2^											
Yes	-	-	-	-	-	-	-	-	-	6,947 (83.4)	6,901 (83.1)
No	-	-	-	-	-	-	-	-	-	1,381 (16.6)	1,408 (16.9)
Missing	-	-	-	-	-	-	-	-	-	0 (0.0)	0 (0.0)
Shift work during the past year^3^											
Yes	15,145 (73.5)	-	-	6,371 (59.8)	6,743 (58.5)	-	4,064 (46.9)	4,395 (42.9)	3,970 (44.7)	3,250 (46.8)	3,009 (43.6)
No	5,466 (26.5)	-	-	4,263 (40.0)	4,781 (41.5)	-	4,594 (53.1)	4,779 (46.6)	4,915 (55.3)	3,697 (53.2)	3,892 (56.4)
Missing	2 (0.0)	-	-	16 (0.2)	3 (0.0)	-	0 (0.0)	1,079 (10.5)	0 (0.0)	0 (0.0)	0 (0.0)
Night shifts during the past year^4^											
Never	1,127 (7.4)	-	-	392 (6.2)	840 (12.5)	-	1,266 (31.2)	1,328 (30.2)	951 (24.0)	912 (28.1)	872 (29.0)
Yes	13,995 (92.4)	-	-	5,979 (93.8)	5,903 (87.5)	-	2,798 (68.8)	3,067 (69.8)	3,019 (76.0)	2,338 (71.9)	2,137 (71.0)
Missing	23 (0.2)	-	-	0 (0.0)	0 (0.0)	-	0 (0.0)	0 (0.0)	0 (0.0)	0 (0.0)	0 (0.0)
Night shifts worked per month^5^	6.59±2.34	-	-	6.22±2.27	6.16±2.18	-	6.01±2.19	5.92±2.01	5.89±2.33	5.72±1.82	5.71±2.12
1-4	1,790 (12.8)	-	-	1,071 (17.9)	1,075 (18.2)	-	588 (21.0)	692 (22.6)	728 (24.1)	542 (23.2)	561 (26.3)
5-7	8,404 (60.1)	-	-	3,756 (62.8)	3,735 (63.3)	-	1,784 (63.8)	1,960 (63.9)	1,930 (63.9)	1,569 (67.1)	1,358 (63.5)
≥8	3,796 (27.1)	-	-	1,152 (19.3)	1,093 (18.5)	-	426 (15.2)	415 (13.5)	361 (12.0)	227 (9.7)	218 (10.2)
Missing	5 (0.0)			0 (0.0)	0 (0.0)		0 (0.0)	0 (0.0)	0 (0.0)	0 (0.0)	0 (0.0)
Alcohol drinking, during the past year											
No	3,479 (16.9)	-	-	-	3,093 (26.8)	-	-	3,025 (29.5)	3,096 (29.1)	-	2,970 (29.0)
Yes	17,104 (83.0)	-	-	-	8,432 (73.1)	-	-	7,228 (70.5)	7,560 (70.9)	-	7,286 (71.0)
Missing	30 (0.1)	-	-	-	2 (0.0)	-	-	0 (0.0)	0 (0.0)	-	0 (0.0)
Currently smoking											
No	20,412 (99.0)	-	-	-	11,441 (99.3)	-	-	10,193 (99.4)	10,595 (99.4)	-	10.196 (99.4)
Yes	197 (1.0)	-	-	-	82 (0.7)	-	-	60 (0.6)	61 (0.6)	-	60 (0.6)
Missing	4 (0.0)		-	-	4 (0.0)	-	-	0 (0.0)	0 (0.0)	-	0 (0.0)
Subjective health (possible range: 1-5)	2.67±0.81				2.63±0.75			2.72±0.77	2.81±0.73	2.86±0.76	2.84±0.75
Missing	3 (0.0)	-	-	-	5 (0.0)	-	-	0 (0.0)	0 (0.0)	0 (0.0)	0 (0.0)

Values are presented as mean±standard deviation or number (%).

1 Excluding the new participants in their 20s who were added (n=1,000).

2 Among the participants who worked.

3 In the 9th survey, among the participants who worked. In the 10th and 11th surveys, among the participants who worked in a health-care facility.

4 Among the participants who had worked shifts during the past year.

5 Among the participants who had worked night shifts during the past year.

**Table 4.** Prevalence of chronic diseases in participants of the KNHS across 11 surveys (2013-2023)

Variables	1st (n=20,613)	2nd (n=15,355)	3rd (n=10,019)	4th (n=10,635)	5th (n=11,525)	6th (n=7,993)	7th (n=8,657)	8th (n=10,253)	9th (n=10,656)	10th (n=10,110)	11th (n=10,256)
Chronic disease											
Hypertension	194 (0.9)	-	74 (0.6)	97 (0.9)	137 (1.2)	-	-	186 (1.8)	235 (2.2)	282 (2.8)	372 (3.6)
Diabetes mellitus	65 (0.3)	-	40 (0.3)	55 (0.5)	63 (0.5)	-	-	87 (0.8)	107 (1.0)	130 (1.3)	149 (1.5)
GERD	3,102 (15.0)	-	1,146 (8.9)	1,807 (17.0)	2,113 (18.3)	-	1,996 (23.1)	1,829 (17.8)	2,043 (19.2)	2,072 (20.5)	2,521 (24.6)
Gastritis/gastric ulcer/duodenal ulcer	3,945 (19.1)	-	1,218 (9.5)	1,969 (18.5)	2,327 (20.2)	-	2,478 (28.6)	1,854 (18.1)	1,880 (17.6)	1,928 (19.1)	2,610 (25.4)
Allergic rhinitis	4,010 (19.5)	-	1,360 (11.0)	1,139 (10.7)	2,429 (21.1)	-	2,280 (26.3)	2,156 (21.0)	2,158 (20.3)	2,293 (22.7)	2,623 (25.6)
Eczema/atopic dermatitis	2,393 (11.6)	-	742 (5.8)	977 (9.2)	1,124 (9.8)	-	939 (10.8)	761 (7.4)	795 (7.5)	867 (8.6)	1,001 (9.8)
Migraine	1,239 (6.0)	-	488 (3.8)	467 (4.4)	699 (6.1)	-	1,142 (13.2)	629 (6.1)	670 (6.3)	728 (7.2)	819 (8.0)
Reproductive disease											
Myoma uteri	904 (4.4)	-	418 (3.3)	635 (6.0)	870 (7.6)	-	879 (10.2)	1,048 (10.2)	1,302 (12.2)	1,399 (13.8)	1,562 (15.2)
Fibrocystic or benign breast disease	694 (3.4)	-	254 (2.0)	486 (4.6)	541 (4.7)	-	-	624 (6.1)	804 (7.5)	862 (8.5)	1,037 (10.1)
Cancer											
Thyroid cancer	148 (0.7)	-	69 (0.5)	107 (1.0)	126 (1.1)	-	107 (1.2)	139 (1.4)	175 (1.6)	183 (1.8)	194 (1.9)
Missing	-		-	-	-		47 (0.5)	-	-	-	-
Breast cancer	29 (0.1)	-	20 (0.2)	29 (0.3)	47 (0.4)	-	36 (0.4)	69 (0.7)	70 (0.7)	76 (0.8)	95 (0.9)
Missing	-		-	-	-		60 (0.7)	-	-	-	-
Cervical cancer	35 (0.2)	-	11 (0.1)	26 (0.2)	38 (0.3)	-	34 (0.4)	81 (0.8)	47 (0.4)	54 (0.5)	56 (0.5)
Missing	-		-	-	-		66 (0.8)	-	-	-	-

Values are presented as number (%); The number of participants shown excludes those who did not provide a response regarding their medical history. KNHS, Korea Nurses’ Health Study; GERD, gastroesophageal reflux disease.

**Table 5.** Characteristics of early-pregnancy and postpartum participants^1^ in the Korea Nurses’ Health Study

Characteristics	Early-pregnancy (n=2,179)	Postpartum (n=2,790)
Age (yr)	32.07±3.68	32.64±3.82
20-29	525 (24.1)	547 (19.6)
30-39	1,582 (72.6)	2,106 (75.5)
≥40	72 (3.3)	137 (4.9)
Planned pregnancy		
Tried hard	951 (43.6)	-
Did not try to conceive	1,219 (55.9)	-
Missing	10 (0.5)	
Pregnancy outcome		
Normal birth	-	2,721 (97.5)
Stillbirth	-	4 (0.1)
Miscarriage	-	51 (1.8)
Abortion	-	6 (0.2)
Ovarian or ectopic pregnancy	-	2 (0.1)
Hydatidiform mole	-	1 (0.0)
Missing	-	5 (0.2)
Current drinking		
No	-	1,849 (66.3)
Yes	-	893 (32.0)
Missing	-	48 (1.7)
Current smoking		
No	2,169 (99.5)	2,730 (97.8)
Yes	2 (0.1)	12 (0.4)
Missing	8 (0.4)	48 (1.7)
Morning sickness		
No	560 (25.7)	-
Yes	1,610 (73.9)	-
Missing	9 (0.4)	-
Vitamin or supplement intake^2^		
Folic acid	1,884 (86.5)	-
Iron	883 (40.5)	-
Beta-carotene	105 (4.8)	-
Fish oil	293 (13.4)	-
Flaxseed or flaxseed oil	69 (3.2)	-
Missing	1 (0.0)	-
Depressive symptom (EPDS)	10.80±5.67^3^	9.29±6.43^4^
Work status		
Yes	1,761 (80.8)	-
No	413 (19.0)	-
Missing	5 (0.2)	-
Shift work^5^		
Yes	841 (47.7)	-
No	920 (52.2)	-
Missing	0 (0.0)	-
Night shifts^6^		
Ever	348 (41.4)	-
Never	493 (58.6)	-
Missing	0 (0.0)	-
Subjective health^7^	2.39±0.79	2.69±0.82^8^
Fatigue (CFS)	13.74±6.26	15.44±5.12^8^
Sleep		
Sleep quality (JSEQ)	6.41±4.79	7.58±4.85^8^
Sleep time (hr)	7.28±1.61	6.36±1.62^8^
Delivery		
Vaginal delivery	-	1,595 (57.2)
Caesarean section	-	1,148 (41.1)
Missing	-	47 (1.7)
Current feeding^2^		
Breast-feeding	-	1,395 (50.0)
Bottle-feeding	-	1,892 (67.8)

Values are presented as mean±standard deviation or number (%). EPDS, Edinburgh Postnatal Depression Scale (possible range: 0-30; ≤9, no symptoms; ≥10, clinical symptoms); CFS, Chalder Fatigue Scale (possible range: 0-33; ≥29, chronic fatigue syndrome); JSEQ, Jenkins Sleep Evaluation Questionnaire (possible range: 0-20; 0-11, few sleep disturbances; 11-20, high frequency of sleep disturbances).

1 The early-pregnancy survey was completed between gestation weeks 20 and 25, and the postpartum survey was completed 6 weeks following the anticipated date of delivery.

2 Multiple choice.

3 n=2,170.

4 n=2,787.

5 Among the participants who worked.

6 Among the participants who worked shifts.

7 Possible range: 1 (not very healthy) to 5 (very healthy).

8 n=2,742.

## DATA ACCESSIBILITY

The initial survey of the KNHS is open to the public through the Clinical and Omics Data Archive (CODA) data repository of the Korea National Institute of Health: https://coda.nih.go.kr/frt/index.do. The successive follow-up surveys will be open to the public in the CODA data repository.
